# Red propolis reduces inflammation in cyclophosphamide-induced hemorrhagic cystitis in rats

**DOI:** 10.7705/biomedica.6087

**Published:** 2022-06-01

**Authors:** Nayanna de Oliveira Ramos Melo, Helio De Sousa Peres Júnior, Clara Araujo Diniz, Matheus De Sousa Silva, Telma Leda Gomes de Lemos, Francisco Vagnaldo Fechine Jamacaru, Conceição Aparecida Dornelas

**Affiliations:** 1 Postgraduate Program in Medical-Surgical Sciences, School of Medicine, Federal University of Ceará, Fortaleza, Brazil Universidade Federal do Ceará School of Medicine Federal University of Ceará Fortaleza Brazil; 2 School of Medicine, Federal University of Ceará, Fortaleza, Brazil Universidade Federal do Ceará Federal University of Ceará Fortaleza Brazil; 3 Biotechnology and Natural Products Laboratory, Department of Organic and Inorganic Chemistry, Federal University of Ceará, Fortaleza, Brazil Universidade Federal do Ceará Biotechnology and Natural Products Laboratory Department of Organic and Inorganic Chemistry Federal University of Ceará Fortaleza Brazil; 4 Laboratory of Pharmacology and Preclinical Research, Nucleus of Research and Development of Medicines, School of Medicine, Federal University of Ceará, Fortaleza, Brazil Universidade Federal do Ceará Laboratory of Pharmacology and Preclinical Research, Nucleus of Research and Development of Medicines School of Medicine Federal University of Ceará Fortaleza Brazil; 5 Postgraduate Program in Pathology and Medical-Surgical Sciences, School of Medicine, Federal University of Ceará, Fortaleza, Brazil Universidade Federal do Ceará School of Medicine Federal University of Ceará Fortaleza Brazil

**Keywords:** Cystitis, cyclophosphamide, propolis, models, animal, cistitis, ciclofosfamida, propóleos, modelos animales

## Abstract

**Introduction.:**

Cyclophosphamide (CP) is used to treat malignant neoplasias and control autoimmune diseases. Still, one of its metabolites, acrolein, is toxic to the urothelium and can lead to hemorrhagic cystitis and severe discomfort.

**Objective.:**

To evaluate the ability of red propolis to prevent and treat CP-induced hemorrhagic cystitis in rats.

**Materials and methods.:**

Red propolis was extracted in 1% gum arabic and administered *subcutaneously* (sc). In the first experiment, groups IA, IIA, and IIIA and groups IB, IIB, and IIIB received water, gum arabic (GA), or propolis, respectively, for 30 days. Then water (controls) or CP (treatment) was administered i.p. In the second experiment, groups IVA, VA, and VIA received water i.p. while groups IVB, VB, and VIB received CP i.p. This was followed by 5 injections at 2-hour intervals with either water, GA, or propolis. Bladder tissue was examined according to Gray’s criteria.

**Results.:**

The total inflammatory histology score was significantly smaller in group VIB (11.33 ± 2.07). Mild inflammation predominated in group VIB while most of the animals in group IVB had severe inflammation (p*=*0.0375). Ulcers were predominantly multiple in Groups IVA and VB but rare or absent in Group VIB (p*=*0.0118). Urothelial cells were mostly absent in groups IVB and VB and present/normal in group VIB (p=0.0052). Fibrin was abundant in groups IVB and VA but mostly absent in group VIB (p=0.0273).

**Conclusions.:**

Red propolis can reduce inflammation in CP-induced hemorrhagic cystitis in rats.

## Resumen

**Introducción.:**

La ciclofosfamida se usa para tratar neoplasias malignas y controlar enfermedades autoinmunitarias, pero uno de sus metabolitos, la acroleína, es tóxico para el urotelio y puede provocar cistitis hemorrágica y malestar grave.

**Objetivo.:**

Evaluar la capacidad del propóleos rojo para prevenir y tratar la cistitis hemorrágica inducida por ciclofosfamida en ratas.

**Materiales y métodos.:**

Se extrajo propóleos rojo en goma arábiga al 1 % y se administró por vía subcutánea. En el primer experimento, los grupos IA, IIA, IIIA, IB, IIB y IIIB recibieron agua, goma arábiga y propóleos, respectivamente, durante 30 días. Luego se les administró agua (controles) o el tratamiento (ciclofosfamida) por inyección intraperitoneal. En el segundo experimento, los grupos IVA, VA, VIA recibieron agua por vía intraperitoneal, y los grupos IVB, VB, VIB recibieron el tratamiento por la misma vía, a lo que le siguieron cinco inyecciones con intervalos de dos horas entre ellas, con agua, goma arábiga o propóleos. El tejido de la vejiga se examinó de acuerdo con los criterios de Gray.

**Resultados.:**

La puntuación total de la inflamación según la histología fue significativamente menor en el grupo VIB (11,33 ± 2,07). La inflamación leve predominó en este grupo, en tanto que la mayoría de los animales del IVB presentó inflamación grave (p=0,0375). Predominaron las úlceras múltiples en los grupos IVA y VB, pero fueron raras o estuvieron ausentes en el VIB (p=0,0118). En general, no se observaron células uroteliales en los grupos IVB y VB, pero sí en el VIB (p=0,0052). La fibrina fue abundante en los grupos IVB y VA, pero predominantemente ausente en el VIB (p=0,0273).

**Conclusiones.:**

El propóleos rojo puede reducir la inflamación en la cistitis hemorrágica inducida por ciclofosfamida en ratas.

The chemotherapy agent cyclophosphamide (CP) is widely used in the treatment of malignant neoplasias such as lymphoma, leukemia, and lung and breast cancer ^1^, and non-neoplastic conditions like systemic lupus erythematosus and rheumatoid arthritis ^2^. However, acrolein, a CP metabolite eliminated in the urine is toxic for urothelial cells by promoting the release of reactive oxygen species (ROS) ^3^. The exposure of the bladder urothelium to acrolein initiates an inflammatory process characterized by edema, leukocyte infiltration, hemorrhage, and ulceration leading to hemorrhagic cystitis and severe suprapubic pain and discomfort ^4^. In the CP-induced cystitis mouse model, CP upregulates c-Fox expression in the spinal cord, increases myeloperoxidase (MPO) activity (an indication of neutrophil accumulation), and promotes proinflammatory cytokines production including interleukin (IL)-1ß and the tumor necrosis factor (TNF)-α ^5^.

Produced by bees (*Apis mellifera*), propolis is known for its wound- healing, anti-inflammatory, antibacterial, antiviral, antifungal, and antitumoral properties. Propolis comes in a variety of compositions and colors (most often red or green) depending on the vegetation in the vicinity of the beehive.

Red propolis has well-documented antimicrobial and antioxidant properties. Brazilian red propolis, one of the 13 types of propolis marketed in the country, is known for its therapeutic potential associated with terpenes, pterocarpans, prenylated benzophenones, flavonoids, and isoflavonoids. This last class of compounds is believed to account for many of its antimicrobial, anti-inflammatory, antioxidant, healing, and antiproliferative effects ^6^.

Brazilian red propolis reportedly has reactive oxygen species (ROS)- scavenging effects *in vitro*. Furthermore, the ethanol extract from Brazilian red propolis (EERP) has been shown to suppress ROS generation and cytotoxicity induced by tert-butyl hydroperoxide. *In vivo*, orally administered EERP increased the expression of Nrf2-regulated genes in mice livers. These results suggest that, as an Nrf2 inducer, EERP is a potential resource for preventing oxidative stress-related diseases ^7^.

Gum arabic (GA) is a natural gum extracted from two sub-Saharan species of *Acacia*. GA at 1% was used to extract red propolis *in natura*, which is insoluble in water ^8^. We chose propolis for this study for its widely acknowledged antioxidant properties ^9^^,^^10^ considering the well-established association between oxidative stress and CP-induced hemorrhagic cystitis ^3^. The purpose of the present study was to evaluate the ability of red propolis to prevent and treat CP-induced cystitis in rats.

## Materials and methods

### Study design

**Figure 1:**
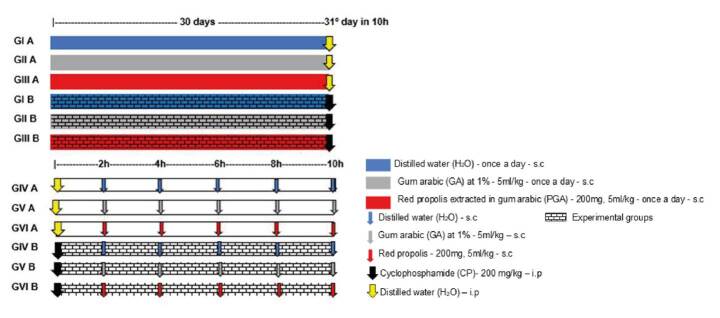
Design of the experiments

To test the potential of red propolis for the prevention of CP-induced cystitis we used 27 female *Wistar* rats distributed in three control groups (n=3) (IA, IIA, IIIA) and three treatment groups (n=6) (IB, IIB, IIIB). Each set of groups received, respectively, distilled water, GA at 1% (5 mL/kg), and red propolis sc (200 mg; 5 mL/kg) once a day for 30 days (figure 1). On the 30th day, distilled water was administered ip in the three control groups while CP (200 mg/kg) was administered ip in the three treatment groups. Ten hours later, the animals were anesthetized and submitted to cystectomy. The bladders were weighed, fixated in 10% buffered formaldehyde, and analyzed according to Gray’s macroscopic and microscopic criteria ^11^.

To test the potential of red propolis for treating CP-induced cystitis we used 30 female *Wistar* rats distributed in three control groups (n=4) (IVA, VA, VIA) and three treatment groups (n=6) (IVB, VB, VIB) (figure 1). All the control groups initially received distilled water ip (5 mL/kg) followed by either distilled water or GA at 1% (5ml/kg) or propolis (200 mg, 5 mL/kg) sc every 2 hours for 10 hours (total: 5 doses). Ten hours later, the animals were anesthetized and submitted to cystectomy. All the treatment groups initially received CP ip (200 mg/kg) followed by either distilled water or GA at 1% (5ml/kg) or propolis (200 mg, 5 mL/kg) sc every 2 hours for 10 hours (total: 5 doses). Ten hours later, the animals were anesthetized and submitted to cystectomy. Finally, the bladders were weighed, fixated in 10% buffered formaldehyde, and analyzed according to Gray’s macroscopic and microscopic criteria ^11^.

Each bladder was embedded in paraffin and examined histologically within 24 hours. Slices (3 μm) were stained with hematoxylin eosine (H&E) and analyzed microscopically according to Gray’s criteria ^11^.

### Materials

Propolis *in natura* was acquired from a trusted supplier in Barra de Santo Antônio (Alagoas). The extraction was performed at the Laboratory of Biotechnology and Natural Products, Department of Organic and Inorganic Chemistry, at the Federal University of Ceará. Insoluble in water, the propolis was extracted with a 1% GA solution as described by Shulka ^8^ and administered sc (200 mg; 5 mL/kg). The antioxidant activity of the red propolis was quantified *in vitro* with a DPPH (1,1-diphenyl-2-picrylhydrazyl) assay ^12^.

Gum arabic 1% AG (Dinâmica Química Contemporânea Ltda.) was diluted in distilled water and administered sc at 5 mL/kg. Cyclophosphamide monohydrate (Genuxal Baxter) was diluted in distilled water using an Esco Isocide laminar flow cabinet and administered ip at 200 mg/kg ^13^ (figure 1).

The bladders were weighed, embedded in paraffin, cut into 3-μm-slices and stained with H&E. Edema, hemorrhage, and histological inflammatory findings were evaluated according to Gray’s criteria ^11^. The following variables were scored:

- Edema: absent=0, mild=1 (amount of fluid between normal and moderate), moderate=2 (fluid observed only on the internal mucosa), severe=3 (fluid on the inside and outside of the bladder wall)
- Hemorrhage: absent=0, mild=1 (telangiectasia/bladder vessel dilation), moderate=2 (mucosal hematomas), severe=3 (intravesical clots)
- Urothelial cells in bladder: present=1 (normal), reduced or absent=2
- Inflammation: absent=0, mild=1, moderate=2, severe=3
- Fibrin deposition: absent=1, present=2
- Submucosal edema: absent=1, mild=2, severe=3
- Ulcers: absent=1, rare=2, multiple=3
- Ulcerations: absent=1, rare=2, multiple=3.

The final histopathological score was the sum of all the above.

### Statistical analysis

The normality of the distribution of discrete and continuous quantitative variables was verified with the Kolmogorov-Smirnov test. The descriptive statistics employed mean values and standard deviations (parametric variables) or medians, minimum and maximum values, and interquartile ranges (non-parametric variables). The three groups of each set were compared pairwise using ANOVA followed by Tukey’s multiple comparison test to detect intergroup differences (parametric variables) or with the Kruskal- Wallis test followed by Dunn’s multiple comparison test (non-parametric variables). The analyses and graphs were made with the software GraphPad Prism v. 5.00 (GraphPad Software, San Diego, California, USA). All tests were two-tailed and the level of statistical significance was set at 5% (p<0.05).

### Ethical considerations

The study protocol complied with the guidelines of the Brazilian Society for Animal Experimentation (BSAE) and was approved by the Committee on Animal Research and Ethics (CARE) of the Federal University of Ceará.

## Results

### Prevention of CP-induced cystitis

**Figure 2. A .**
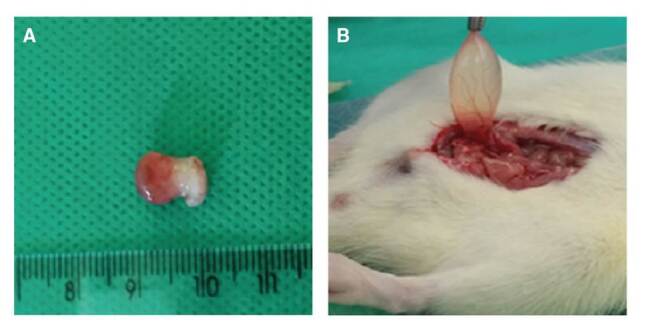
Swollen bladder with telangiectasia from group IB (treatment). **B.** Bladder from Group IA (control)

**Figure 3.**
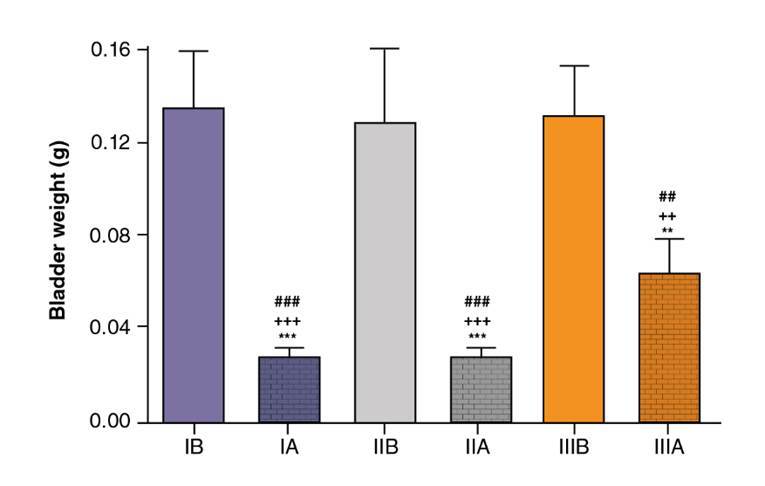
Mean bladder weight (g) in groups IA, IB, IIA, IIB, IIIA, and IIIB

The bladder weight (mean ± standard deviation) was smaller in groups IA, IIA, and IIIA than in groups IB, IIB, and IIIB (figures 2 and 3). The total histological scores (mean ± standard deviation) of groups IB (14.33 ± 1.63), IIB (14.17 ± 1.17), and IIIB (13.67 ± 1.03) were statistically similar (ANOVA: F=0.4248; p*=*0.6615). The pattern of inflammation, ulceration, hemorrhage, and histological changes was statistically similar for groups IB, IIB, and IIIB (p=0.1828, p=0.3303, p=0.2070 and p=0.4066, respectively).

### Treatment of CP-induced cystitis

**Figure 4.**
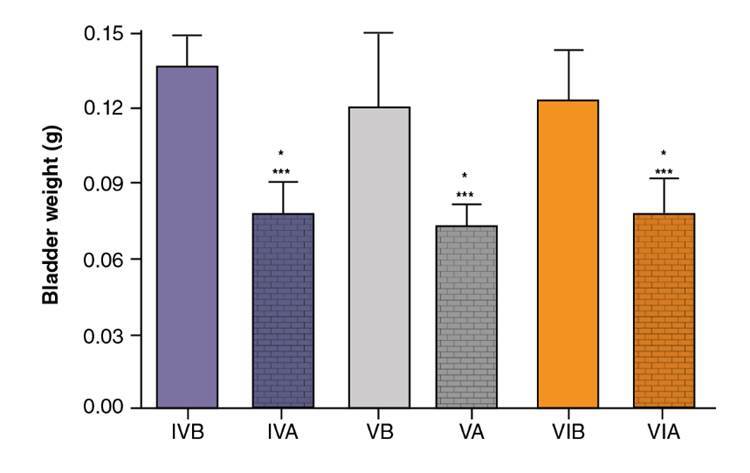
Mean bladder weight (g) in groups IVA, IVB, VA, VB, VIA, and VIB

**Figure 5.**
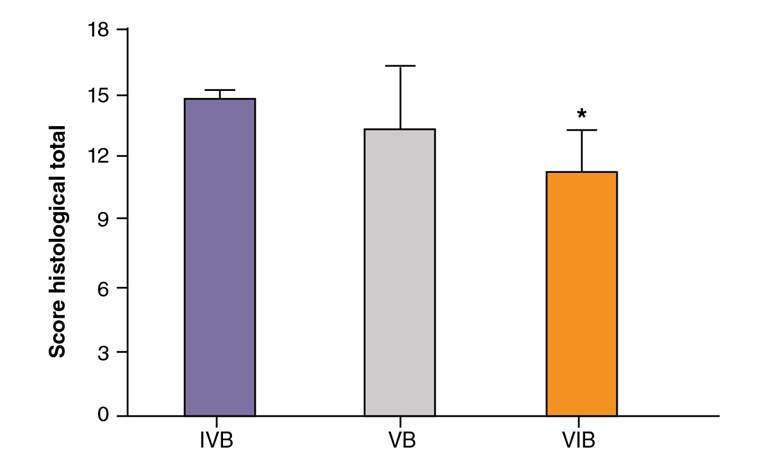
Total histological scores (mean ± standard deviation) in groups IVB, VB, and VIB according to Gray’s criteria

The bladder weight (mean ± standard deviation) was smaller in groups IVA, VA, and VIA than in groups IVB and VIB (figure 4). The total histological score (mean ± standard deviation) was significantly smaller in group VIB (11.33 ± 2.07) than in group IVB (14.83 ± 0.41), but the score of group VB (13.50 ± 2.81) did not differ significantly from that of the other two groups (*p*>0.05) (figure 5).

Groups IVB, VB, and VIB differed significantly with regard to the presence of urothelial cells in the bladder (p*=*0.0052). All or most of the bladders in groups IVB (100%) and VB (83.33%) were devoid of urothelial cells while the number of cells was classified as normal in most of the bladders in group VIB (83.33%) (table 1).

**Table 1:** Presence of urothelial cells in bladders from groups IVB, VB, and VIB

Group	Urothelial cells		Total
	Present (normal)	Absent	
IVB	0 (0.00%)	6 (100%)	6 (100.00%)
VB	1 (16.67%)	5 (83.33%)	6 (100.00%)
VIB	5 (83.33%)	1 (16.67%)	6 (100.00%)
Total	6	12	18

Chi-squared test

Groups IVB, VB, and VIB differed significantly with regard to the level of inflammation (p*=*0.0375). Mild inflammation was predominant in group VIB (83.33%) while most of the animals in group IVB had severe inflammation (83.33%) (table 2).

**Table 2:** Level of inflammation in groups IVB, VB and VIB

Group	Inflammation			Total
	Mild	Moderate	Severe	
IVB	0 (0.00%)	1 (16.67%)	5 (83.33%)	6 (100.00%)
VB	2 (33.33%)	1 (16.67%)	3 (50.00%)	6 (100.00%)
VIB	5 (83.33%)	1 (16.67%)	0 (0.00%)	6 (100.00%)
Total	7	3	8	18

Chi-squared test

Groups IVB, VB, and VIB also differed significantly with regard to the presence of ulcers (p*=*0.0118). Multiple ulcers were the predominant finding in groups IVA (100%) and VB (83.33%) while they were generally rare or absent in group VIB (83.33%) (table 3).

**Table 3:** Presence of ulcers in Groups IVB, VB, and VIB

Group	Ulcers		Total
	Absent/rare	Multiple	
IVB	0 (0.00%)	6 (100.00%)	6 (100.00%)
VB	2 (33.33%)	4 (66.67%)	6 (100.00%)
VIB	5 (83.33%)	1 (16.67%)	6 (100.00%)
Total	7	11	18

Chi-squared test

The level of edema was statistically similar in groups IVB, VB, and VIB (p*=*0.5698) but the difference was significant with regard to fibrin deposition (p*=*0.0273). Thus, fibrin was observed in almost all the bladders in groups IVB (100%) and VA (83.33%) but was predominantly absent in group VIB (66.67%) (table 4).

**Table 4:** Presence of fibrin in groups IVB, VB, and VIB

Group	Fibrin		Total
	Absent	Present	
IVB	0 (0.00%)	6 (100.00%)	6 (100.00%)
VB	1 (16.67%)	5 (83.33%)	6 (100.00%)
VIB	4 (66.67%)	2 (33.33%)	6 (100.00%)
Total	5	13	18

Chi-squared test

**Figure 6.**
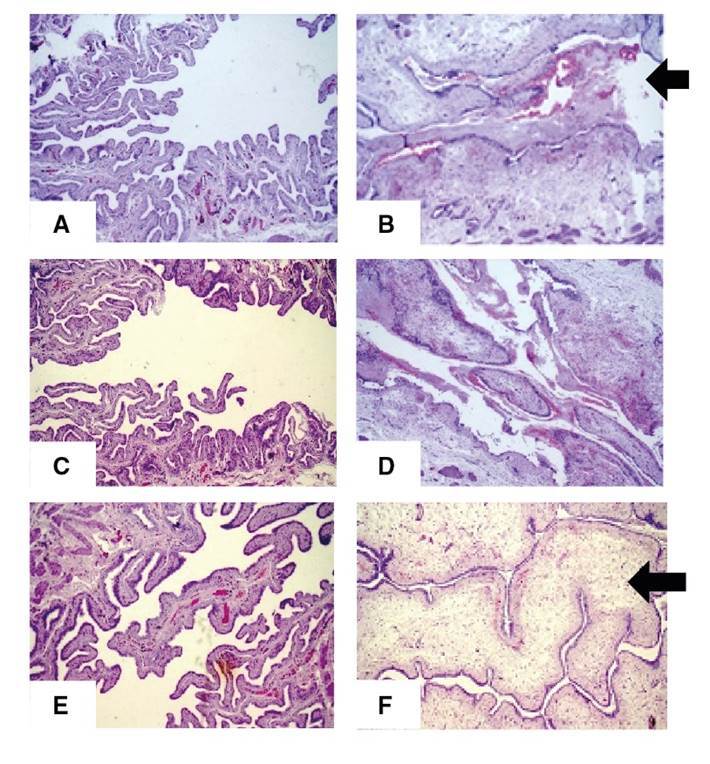
Slices of bladder wall stained with H&E (magnification: 100X). **A.** Group IVA; normal aspect. **B.** Group IVB; edema and inflammatory infiltrate in the submucosa and large amount of fibrin and red blood cells in the bladder lumen (black arrow). **C.** Group VA; normal aspect. **D.** Group VB; inflammatory infiltrate in the submucosa and large amount of fibrin and red blood cells in the bladder lumen. **E.** Group VIA; normal aspect. **F.** Group VIB; inflammatory infiltrate in the submucosa and fibrin and red blood cells in the bladder lumen significantly reduced in relation to Group IVB (B), despite accentuated edema in the submucosa (white arrow).

Finally, the three treatment groups (IVB, VB, and VIB) displayed statistically similar results for hemorrhage (p*=*0.1847) and marginally significant differences with regard to histological changes (p*=*0.0537) (table 5; figures 6A-F).

**Table 5:** Histological change in groups IVB, VB, and VIB

Group	Histological change		Total
	Normal/mild	Severe	
IVB	1	5	6
VB	2	4	6
VIB	5	1	6
Total	8	10	18

Chi-squared test

## Discussion

CP is an oxazaphosphorine alkylating agent used as a broad-spectrum antineoplastic agent and as immunosuppressant in non-neoplastic conditions like systemic lupus erythematosus and rheumatoid arthritis. Like other prodrugs, CP is converted by mixed-function oxidase enzymes (cytochrome P450 system) in the liver to form active metabolites. The metabolites phosphoramide mustard and acrolein are responsible for the induction of oxidative stress ^14^ but can also promote cystitis, pneumonitis, pulmonary fibrosis ^15^, and genotoxicity ^16^. Damage to the urothelium with CP is caused by acrolein. Urothelial injury has been shown to be directly associated with acrolein inducing necrosis, edema, ulceration, hemorrhage, and leukocyte infiltration and, at a later stage, fibrosis ^17^.

Due to the usefulness of CP in the control and treatment of severe and prevalent conditions, the search for compounds capable of preventing and reverting tissue damage is of utmost importance. Much research has relied on animal models, one of which is the mouse/rat model of CP-induced hemorrhagic cystitis. In this study, we administered a single dose of 200 mg per kg of body weight ip as described by Maia, *et al.*^13^, but other doses have been tested. As expected, we observed neutrophil infiltrate approximately six hours after the inflammatory challenge, apparently associated with the presence of acrolein released by the liver 30 min after the administration of CP ip ^18^. The histological analysis confirmed inflammation as initially inferred from the increase in bladder weight. By the end of the 10-hour period, the level of acute inflammation had significantly increased in relation to controls. The CP-induced hemorrhagic cystitis model used in this study was efficient at evaluating both preventive and therapeutic effects, as shown in figures 3 and 4.

In 2012, Ribeiro, *et al*. described four distinct stages of acrolein-induced bladder inflammation: In the first stage, the metabolite is concentrated in the bladder epithelium. The second stage is characterized by inflammation as epithelial and connective tissue cells (including macrophages) promote a local increase in inflammatory cytokines (especially TNF-α and IL-1). An increase is also seen in ROS and in the expression of inflammatory enzymes (such as inducible nitric oxide synthase and cycloxigenase-2). In the third and ulcerative stage, epithelial cells are lost and ulcers develop causing lower urinary tract symptoms and bladder dysfunction. Finally, in the fourth stage, tissue repair initiates, possibly by way of fibroblast signaling and local increase in growth factors (such as keratinocyte growth factor) ^19^.

Several compounds have been tested experimentally to prevent or treat CP-induced toxicity. This includes an interesting study evaluating the potential of Egyptian propolis to protect against liver and kidney injury in mice with promising results in terms of toxicity and immunosuppression ^20^.

The variation observed in the color (black, yellow, brown, green, red) and composition of propolis depends on the vegetation found in the vicinity of the beehive ^21^. Differences in composition are reflected in pharmacological properties, a phenomenon more easily studied in tropical regions ^22^. The red propolis employed in this study was produced in the State of Alagoas (Northeastern Brazil) based on exudate from the coinvine (*Dalbergia ecastophyllum*), a legume rich in flavonoids.

Recently, upon extraction of red propolis in ethanol at our laboratory, six compounds were identified: i) 2’-hydroxy-4,’7-dimethoxy-isoflavane, ii) 2,’7-dihydroxy-4’-methoxy-isoflavane, iii) 2,’4’-dihydroxy-7-methoxy-isoflavane, iv) 4,’7-dihydroxy-2’-methoxy-isoflavane, v) 2,’4,’4-trihydroxy-chalcone, and vi) lup-20(29)-en-3-ol. The extract and its fractions were submitted to DPPH antioxidant activity assays. At a concentration of 1 mg/mL, activity was significant (99.8% inhibition) for the ethanolic extract, the ethyl acetate fraction, and the methanol fraction. All three yielded significant IC_50_ values and performed better than the vitamin C standard ^12^.

Also, our team tested the ability of red propolis treatment for 16 weeks to reduce oxidative stress based on TBARS quantification in peripheral blood of rats with azoxymethane-induced colon cancer ^23^. The development of the present experimental study was motivated by the results of *in vitro* and *in vivo* studies of the pathophysiology of CP-induced hemorrhagic cystitis and its association with oxidative stress ^19^.

In this study, the administration of 5 doses of red propolis extract at 2-hour intervals as treatment for CP-induced cystitis significantly reduced the total score of inflammatory changes on histology ^11^ (figure 5). Treatment with propolis also protected the urothelial epithelium (table 1) by reducing the level of inflammation (table 2) and the incidence of ulcers when compared to epithelia from animals treated with CP only (table 3) and reduced the incidence of fibrin deposition (table 4), but no measurable effect was observed in the level of edema and hemorrhage. In other words, the reduction in inflammation observed biologically using an animal model was compatible with the results of earlier studies evaluating the effect of propolis *in vitro*.

Propolis from Turkey and Egypt is reported to contain caffeic acid phenethyl ester (CAPE). This compound, which has also been identified in Brazilian propolis (e.g., green propolis), is known to reduce oxidative stress, block the production of ROS in human neutrophils and xanthine oxidase systems ^24^, and protect against hemorrhagic cystitis in rats treated with CP by lowering malondialdehyde levels in the bladder. When administered before CP, CAPE reduced the concentration of nitric oxide, which plays an important role in the pathophysiology of CP-induced hemorrhagic cystitis ^25^.

In earlier experiments on animal models conducted by our team, red propolis inhibited inflammatory and neoplastic angiogenesis associated with a reduction in inflammatory cells (e.g., mastocytes and macrophages) suggestive of immunomodulatory action ^26^^,^^27^.

When evaluating the potential of red propolis to prevent cystitis (using a single daily injection of propolis or GA sc for 30 days prior to the administration of CP), the level of edema, hemorrhage, ulcers, and inflammation was not significantly reduced, as shown by the staining with H&E. Much attention has been given to the immunological and immunomodulatory properties of red and green propolis in the prevention of inflammation and neoplasia but with the regime adopted in this study no such effect was observed for CP-induced cystitis. Nevertheless, other regimes might yield different results. 

When administered sc as treatment for hemorrhagic cystitis in rats, red propolis reduced inflammation and the total score of histopathological changes, and protected the bladder epithelium by reducing fibrin deposition, inflammatory cells, and ulcers.
